# Thiolation of Biopolymers for Developing Drug Delivery Systems with Enhanced Mechanical and Mucoadhesive Properties: A Review

**DOI:** 10.3390/polym12081803

**Published:** 2020-08-11

**Authors:** Vivek Puri, Ameya Sharma, Pradeep Kumar, Inderbir Singh

**Affiliations:** 1 Chitkara College of Pharmacy, Chitkara University, Punjab 140401, India; vivek.puri@chitkara.edu.in (V.P.); ameya.sharma@chitkara.edu.in (A.S.); 2 Chitkara University School of Pharmacy, Chitkara University, Himachal Pradesh 174103, India; 3 Department of Pharmacy and Pharmacology, School of Therapeutic Sciences, Faculty of Health Sciences, University of the Witwatersrand, Johannesburg 2193, South Africa; pradeep.kumar@wits.ac.za

**Keywords:** thiolation, biopolymers, drug delivery systems, mechanical properties, mucoadhesive properties, patents

## Abstract

Biopolymers are extensively used for developing drug delivery systems as they are easily available, economical, readily modified, nontoxic, biodegradable and biocompatible. Thiolation is a well reported approach for enhancing mucoadhesive and mechanical properties of polymers. In the present review article, for the modification of biopolymers different thiolation methods and evaluation/characterization techniques have been discussed in detail. Reported literature on thiolated biopolymers with enhanced mechanical and mucoadhesive properties has been presented conspicuously in text as well as in tabular form. Patents filed by researchers on thiolated polymers have also been presented. In conclusion, thiolation is an easily reproducible and efficient method for customization of mucoadhesive and mechanical properties of biopolymers for drug delivery applications.

## 1. Introduction

Biopolymers are the polymers of biological origin and biocompatible/biodegradable. They are also known as bio-polymeric molecules, bio-molecules, biomaterials. Chemically, they are monomeric units, conjugated by covalent bonds to form large structures. Structurally, they are renewable materials and also sustainable as they are acquired biologically (plants). Biopolymers also reveal significant property as epitomize; biodegradable in nature, biologically compatible and bacterial resistant activity [1,2]. According to monomeric unit and structurally, biopolymers are diversified into three main classes named as polynucleotides (long polymers); RNA and DNA, are comprised of 13 or more monomers (nucleotide), polypeptides (short polymers), consist of amino acids and lastly, the polysaccharides are polymeric carbohydrate structures which are linearly bonded. They occur in our environment and can be easily extracted, foremost these are both soluble and insoluble in water [3]. 

The phenomenon shown by two substances when forced together for a long duration by interfacial force is called adhesion. Mucoadhesion is generally observed between polymers and epithelial/mucosal surfaces [4]. Controlled drug delivery systems optimize the pharmacokinetics and pharmacodynamics of a drug molecule and increase its efficiency by decreasing its side effects and providing a faster treatment via the most suitable route. Mucoadhesive dosage forms target local areas of mucosal surface at a lower dose, the surface area of mucosal surfaces in the body being a huge factor in the adhesion of various drugs. The area of mucosal surfaces is larger than skin and hence it proved to be 4 times more effective than that of skin [5,6]. Polymers play a vital role for the development of mucoadhesive dosage form. Both the polymers (natural and synthetic) exhibit extensively higher mucoadhesive properties, and the synthetic polymers shows their actions and retain the drug for a longer duration in the body on contrast to natural polymers. However, the modification (thiolation) of synthetic polymers is done in barren conditions as compared to natural polymers and they tend to be less biodegradable in comparison [7,8]. Some examples of biopolymers with respect to origin, chemical structure and properties which are mainly used for modification to enhance the mucoadhesive properties are illustrated in Table 1. Polymers used for thiolation tend to be hydrophilic because of their high water solubility and therefore there are more targeting sites for the formation of ‘bonds (hydrogen with mucin)’ which result in greater adhesion. Such thiolation of mucoadhesive polymers leads to increase in the mucoadhesion by 140 folds, this occurs because of spontaneous formation of intra- and inter-disulfide bonds with mucosal layer (cysteine rich areas) [9,10]. Mucoadhesive controlled release drug delivery systems offers advantages as compared to the standard drug delivery system. These advantages are; (i) site specific drug delivery, (ii) less side effects, (iii) more contact time between drug and surface, (iv) controlled/slow drug release, (v) improved bioavailability. A polymer is a macromolecule that consists of smaller subunits called monomers. They are of two types natural and synthetic. Polymers are used in dry forms and they are water soluble so they form a strong interaction with the mucosal layer e.g., implants, beads and nano-particles as listed in Table 2 [11,12].

Thiomers (thiolated polymers), consisting of thiol groups and having side chains are foremost and significant mucoadhesive polymers. These are used extensively in the development of various drug delivery systems (therapeutic drug delivery systems) and have extensive advantages over other polymer based systems [29]. Thiomers exhibit improved cohesive properties, as they form inter and intra chain bonds (disulfide) that leads to the greater mucoadhesiveness and formation of disulfide bonds between cysteine rich domains of glycoproteins and the polymers [30,31].

Thiomers are classified on the basis of their parent chemical molecule [32]

(1) Cationic thiomers—the glucosamine contain a primary amino group at the second position that acts as the primary target for thiol group immobilization. The link between the two is formed covalently and the bond could either be amine or amidine. If the bond is amine, the carboxylic group of cysteine and thioglycolic acid reacts with the primary amine site of polymer. In case of amidine bond, 2-imidothiolane acts as a coupling agent. The advantage of using this is that the reaction occurs in a single step.
(2) Anionic thiomers—these types of thiomers have carboxylic acid groups as the anionic target. They are advantageous as sulfhydryl moieties can easily attach to cysteine and homocysteine ligands with the help of amide linkage too. These bonds are mediated by carbodiamides.

Thiolated polymers or thiomers contain free thiol groups on the polymer moiety that causes an increase in mucoadhesion as compared to normal polymers. The thiol groups allow the formation of strong disulfide bonds between the mucin surface and the thiolated polymer. These are mostly used in controlled drug delivery systems [33]. The more thiol groups present on the polymer backbone, the more cohesive and adhesive will be the polymer. Unbound moiety will decrease adhesion to some extent. Mucoadhesive polymers are non-toxic, not absorbed in the gastrointestinal tract (GIT), prolongs the residence time, non-irritant to mucous membrane, possess site-specificity and form a strong covalent bond with the mucous membrane [34,35]. Duggan et al. demonstrated thiolated polyallylamine to be a promising mucoadhesive polymer. The polymer was reported to possess high swelling and cohesive properties [36]. Kazemi et al. developed thiolated chitosan-lauric acid as a new derivative of chitosan for drug delivery and biomedical applications [37]. Griesser et al. reported thiolated hyaluronic acid to be a versatile polymer for drug delivery and biomedical application with improved properties viz. gelling, mucoadhesion, permeation enhancement, enzyme inhibition and controlled/sustained drug release [38].

Natural polymers are an important part of pharmaceutical formulations because they are biocompatible with various substances and can be easily extracted. Examples include, but not limited to, Guar gum, Okara gum, Moringa gum, Locust bean gum, Chitosan, Alginate and Xanthan gum [39]. Patents filed by researchers on thiolated biopolymers are discussed in Table 3.

**Figure 1:**
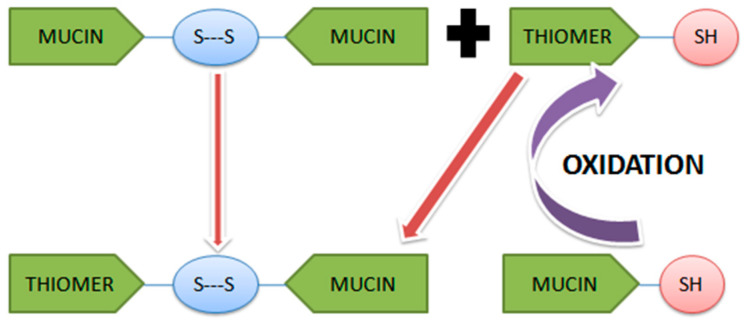
Mechanism of formation of disulfide bond (-SH) between thiomer and mucin.

Mucin is having cysteine rich domains, present all over the mucus membrane. Formation of disulfide bonds with the thiomer depends upon the interaction of thiol groups with these cysteine groups of mucin either by oxidation of the thiol groups or by thiol/disulfide exchange reaction [30] as illustrated in Figure 1.

Mucoadhesion takes place depending upon the pH of thiolated polymer, pH of the surrounding medium and pKa of the thiol group. The disulfide bonds thus formed are not influenced by the ionic strength and pH condition [5].

## 2. Methods of Thiolation

### 2.1. Thiolation by Traut’s Reagent

**Figure 2:**
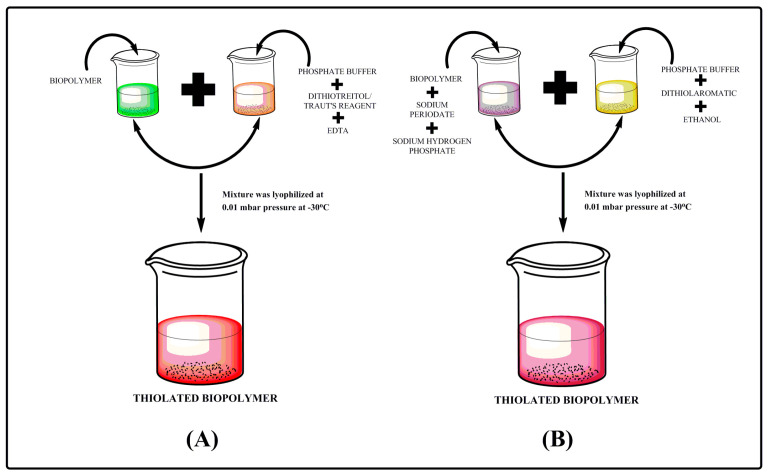
Step wise process of thiolation (**A**) by Traut’sreagent/dithiotreitol and (**B)** by dithiolaromatic.

Traut’s reagent; chemically known as 2-iminothiolane is a cyclic compound which is widely used for thiolation reactions. Traut’s reagent acts with primary amines to introduce sulfhydryl group while keeping all charge properties constant. These can be then used for cross linking or immobilization reactions. As reported in literature, for thiolation procedure (Figure 2), biopolymer was dissolved in a non-amine buffer (pH 7.4) and Traut’s reagent and EDTA were added in quantity that depends on the protein size and concentration of the biopolymer. The reaction mixture was then incubated at room temperature for 1 h. The unreacted reagent was filtered and the presence of sulfhydryl groups was determined by using Ellman’s reagent [58,59].

### 2.2. Thiolation by Dithiothreitol (DTT) Reduction

DTT is the trans isomer of compound 2,3-dihydroxy-1,4-dithiolbutane. It is able to reduce all biological sulfhydryl groups and to maintain free thiol groups despite the presence of oxygen. Biopolymer was dissolved in phosphate buffer pH 7.4. The quantity of DTT and EDTA depends upon the concentration and quantity of the biopolymer. The preparation was reacted at room temperature for 1 h, presented in Figure 2. An unreacted reagent was filtered and the sulfhydryl groups were checked by using Ellman’s reagent [58,60,61].

### 2.3. Thiolation by Dithiolaromatic (PEG6-CONHNH_2_)

Dithiol aromatic is a compound that displaces and increases the number of cysteine residues in turn resulting in formation of non-native disulfide bonds. To carry out the experiment; the biopolymer was mixed with sodium hydrogen phosphate and sodium periodate and kept in the dark for 30 min. Mixtures of phosphate buffer solution, dithiol aromatic and ethanol was added to the above prepared solution. The preparation was reacted at room temperature for 1 h; Figure 2. Finally, the unreacted reagent was filtered and Ellman’s method was used to determine the presence of sulfhydryl groups on thiolated biopolymer [58,62].

### 2.4. Thiolation by Thiol Polyethylene Glycolamine (SH-PEG-NH_2_)

**Figure 3:**
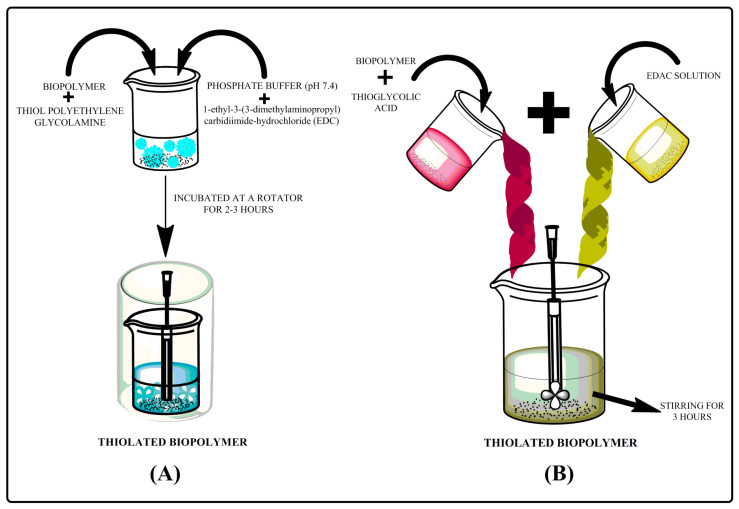
Step wise process of thiolation (**A**) by thiol polyethylene glycolamine and (**B**) by thioglycolic acid.

Biopolymer was added to thiol polyethylene glycolamine, phosphate buffer pH 7.4 and EDAC or 1-thyl-3-(3-dimethylaminopropyl) carbodiimide-hydrochloride and this mixture was incubated on a rotator for about 2 h, as presented in Figure 3. An unreacted compounds were filtered and Ellman’s reagent was used to determine the presence of sulfhydryl groups (-SH) present over thiolated biopolymer [58,63].

### 2.5. Thiolation by Thioglycholic Acid

Ethanolic extract of biopolymer was dissolved in thioglycolic acid (TGA) and EDAC or 1-thyl-3-(3-dimethylaminopropyl) carbodiimide-hydrochloride was added to the prepared mixture slowly with constant stirring for about 3 h as illustrated in Figure 3. Unreacted compound was filtered and Ellman’s reagent was used to determine the sulfhydryl groups (-SH) [58,64].

## 3. Characterization

The characterization of biopolymer is determined by various methods which include thiol group content using iodometry titration and Ellman’s method. Further, disulfide group content, swelling index and mucin interaction with polymer are also measured in different media.

### 3.1. Determination of Thiol Group Content

#### 3.1.1. Determination of Thiol Group Content by Iodometry Titration Method 

The polymer thiol group content was determined by iodometry. Thiolated biopolymer was added in the iodine flask for hydrating it with demineralized water. The pH was adjusted by adding 1M HCl in the solution and then the standard solution (0.1 N iodine) was also added and shook for 30 min. Disproportionate amount of iodine in the solution was titrated with 0.1 N sodium thiosulfate solution using starch as an indicator [65]. Furthermore, Barbaric et al. performed iodometric titration to determine the free SH group present on thiolatedpolyaspartamide and it was concluded that around 6.9 to 45.6 µmol·g^−1^ amount of free SH groups were immobilized on the backbone of thiolatedpolyaspartamide [66]. Additionally, Bernkop-Schnürch et al. revealed the determination of thiol content via iodometry titration method and was found to be 148 ± 42 µmol of SH group per gram of polymer [67].

$$\%\;\mathrm{Thiol}\;\mathrm{group}\;\mathrm{content}=\frac{\left[\left(\mathrm{Blank}-\mathrm{Proper}\right)\times 0.1\times 0.066\times 100\right]}{0.1}\times \;\mathrm{weight}\;\mathrm{of}\;\mathrm{thiomer}$$

#### 3.1.2. Determination of Thiol Group Content by Ellman’s Method 

The degree of thiol group content was determined by employing Ellman’s method. This method was executed by quantifying the amount of thiol group, thiolated biopolymer solutions were prepared in 1N NaOH and diluted with an equal volume of phosphate buffer having pH 8.0. This prepared solution was further allowed to react with Ellman’s reagent (DTNB) in phosphate buffer with pH 8.0 at room temperature for 2 h. The thiol group substitution of reaction mixture was determined by measuring absorbance at 450 nm and the numbers of thiol groups present in thiolated biopolymer were calculated using the standard curve plotted by reacting the native polymer solution containing varying amount of thiolated reagent with Ellman’s reagent [28,30]. Researchers synthesized thiolated pectin and further determined the presence of thiol group in one gram of polymer (0.60 ± 0.04 mM) by employing Ellman’s method. Furthermore, in his another study he and his team synthesized thiolated moringa gum and determination of thiol content was evaluated by using Ellman’s reagent and was found to be 0.956 ± 0.024 mM of thiol groups per gram of polymer [28,68].

### 3.2. Disulfide Bond Formation 

In a round bottom flask (RBF), thiomer was hydrated with iodine and pH (2–3) range was adjusted by adding 1M HCl. Then, 3% solution of sodium borohydride was added to the polymer solution and stirred for 15–20 mins to hydrate all the disulfide bonds to free thiol groups. Finally, 1M HCl was added to neutralize the mixture and the estimation of disulfide content was done by subtracting the thiomer before and after reduction of thiol groups [65,69,70].

$$\%\;\mathrm{Disulfide}\;\mathrm{group}\;\mathrm{content}=\frac{\left[(B_{1}-{\;B}_{2})\times 0.1\times 0.066\times 100\right]}{0.1}\times \;\mathrm{weight}\;\mathrm{of}\;\mathrm{thiomer}$$

where, B_1_ = Blank, burette reading (after reduction); B_2_ = Blank, burette reading (before reduction).

Bernkop-Schnürch et al. developed controlled release systems using thiolated polymers and performed disulfide bond formation within the polymer conjugate and revealed that at pH 6.8 there was a formation of disulfide bridge in comparison to pH 5.0. Apart from all this, formation of disulfide bonds mostly depends upon the concentration of thiomers. The higher the polymer-cysteine conjugate concentration is, the more disulfide bonds will be formed. Furthermore, they concluded that disulfide bonds formed within a matrix-tablet comprising thiolated polymer provide higher stability and higher viscoelastic properties [71]. In another study, Bernkop-Schnürch et al. fabricated microparticles of poly(acrylic acid)-cysteine conjugates and revealed that microparticles prepared at pH 9 show higher concentration of thiol group and higher mucoadhesion properties on contrast to microparticles prepared at pH 3 and pH 6 [72]. Schmitz et al. revealed that chitosan-N-acetyl conjugate was more stable with decrease in amount of thiol group by 40% at pH 5 in comparison to pH 6 (rapid decrease in thiol group by 65%) [73].

### 3.3. Swelling Behavior

Evaluation of swelling behavior could be used for comparing pure and thiolated polymers. Polymers could be compressed into discs of specified dimensions keeping pressure and other parameters as constant. Prepared discs can be used for calculating swelling index in media of defined pH [65,74,75].

$$\mathrm{Swelling}\;\mathrm{index}=\frac{W_{2}-W_{1}}{W_{1}}\times 100$$

where, W_1_ = initial weight of tablet, W_2_ = final weight of tablet (after dipped in demineralized water).

Bernkop-Schnürch et al. examined the swelling behavior of matrix-tablet and concluded that there is no significant change in swelling behavior of tablet prepared with thiolated carboxymethyl cellulose and the native polymer. However, thiolated polycarbophil showed decrease in water uptake when compared with unmodified polycarbophil [71]. Additionally, one more study revealed the swelling behavior of thiolated polymer (chitosan). In this the author employed student *t*-test (*p* > 0.05) and concluded no significant increase in swelling behavior of the thiolated polymer with respect to unmodified polymer [72]. 

### 3.4. Evaluation of Mucoadhesion

Mucoadhesion or bioadhesion study was evaluatedby mucin adsorption study using calorimetry method. Further, polymer mucin interaction study was based on the viscosity of the solution.

#### 3.4.1. Atomic Force Microscopy

This method is used to perform bioadhesion studies based on the changes occur on the rough surface of the polymer after binding to a biological tissue. Bonds formed between the polymer and the tissue lead to higher surface roughness. Atomic force microscopy (AFM) is used to study the force as well as surface properties which are necessary to remove the polymer or adhesive formulation from a tissue. The bioadhesive force between Pluronic–poly(acrylic acid)copolymer and mucin-coated surface was determined by Cleary and his co-workers. Furthermore, they concluded that the changes in various (pretest and withdrawal) speed and measurement time have a considerable influence on the mucoadhesion or bioadhesion forces [76]. Cleary et al. performed atomic force microscopic method for investigating the interaction between Pluronic–poly(acrylic acid)-modified microsphere and mucous substrates. Atomic force microscopic images of unmodified slide showed roughness average (R_a_ = 8.1 nm) with smooth and flat surface in comparison to Pluronic–poly(acrylic acid)-modified microsphere slide imaged in air (R_a_ = 21.8 nm), and Pluronic–poly(acrylic acid)-modified microsphere slide imaged in phosphate buffer solution (pH 7.0) (R_a_ = 61.5 nm). In conclusion, they revealed that as the mucin was immersed in buffer solution, the mucin got swell and further lead to increase in R_a_ value [77].

#### 3.4.2. Mucin Adsorption Study

Mucin adsorption study can be performed by mucous glycoprotein assay method which employs periodic acid/shift (PAS) calorimetric method for computing mucin concentrations which in turn can be used for calculating the amount of mucin adsorbed by the polymer and/or the formulation. Mucin adsorption data can be further fitted to Freundlich and Langmuir equations for assessing the involvement of electrostatic forces in the process of adsorption and interaction of mucin with the polymer or the drug delivery system [78]. Atyabi et al. formulated thiolated chitosan-amikacin conjugate nanoparticles for oral drug delivery. The evaluation for mucin glycoprotein assay was done to determine the free mucin and mucin adsorbed on the thiomers. Furthermore, this evaluation was performed to check the effect on mucoadhesive behavior of thiomers. Based on the result, he concluded that there is a strong attraction between the mucin and chitosan which resulted in the slightly increase in the mucoadhesive properties of N-acetyl cysteine (NAC) and N-acetyl D-penicillamine (NAP) chitosan conjugates in comparison to cysteine-chitosan conjugate. This was because of the hydrophobic interaction between the polymer acetyl groups and mucin. There was occurrence of higher attraction for mucin in conjugates (NAP-chitosan). Comparatively, the structures of two conjugates represent that NAP has two methyl groups additionally. Furthermore, it has higher mucoadhesive properties due to interaction (stearic and hydrophobic) of methyl groups with mucin [79].

#### 3.4.3. Polymer–Mucin Interaction Study

Mucus layer consist of water (95% by weight), mucin (NMT 5% by weight), inorganic salts (=1% by weight), carbohydrates and lipids as main components. Mucin represents more than 80% of the organic components of mucus and is responsible for gel like consistency of the mucus. Chemically, mucin is composed of galactose, N-acetylgalactosamine, N-acetylglucosamine, sialic acid and fucose. Interaction between mucoadhesive polymer and mucin results in changes in the rheological behavior. Hence, viscosity of invitro molecular dispersion of polymer and mucin could be correlated with strength of mucoadhesion. Thus, viscosity synergism could be regarded as an in vitro parameter for measuring and comparing the mucoadhesive properties of various polymers. Greater viscosity synergism could be regarded as an indicative of stronger polymer–mucin interaction. Viscometer could be used for performing viscosity measurement of pristine polymer and mucin and the same in different proportions [80].

η_exp_ = η_p_ + η_m_

η_enhance_ = η_obs_ − η_exp_

η_rel_ = η_obs_/η_exp_

where, η_exp_ = expected viscosity, η_obs_ = observed viscosity, η_enhance_ = viscosity enhancement, η_rel_ = relative viscosity.

Viscosity component of bioadhesion (η_b_) can be calculated as:

η_t_ = η_m_ + η_p_ + η_b_

where, η_t_ = viscosity of the system, η_m_ = viscosity of mucin, η_p_ = viscosity of polymer.

Thirawong et al. performed viscometric study of pectin-mucin interaction on different grades of pectin (CU201, CU501, CU701 and CU020), Carbopol 934P and chitosan (low and medium molecular weight) using Brookfield viscometer and concluded that the viscosity of pectin (CU701) was increased with increase in ionic strength. Nevertheless, the addition of glucose leads to increased viscosity of all pectins. The blended combination of pectin and mucin in fluids such as simulated gastric fluid (SGF), simulated intestinal fluid (SIF) and deionized water (DI) water exhibited increased viscosity as compared to polymer having synergistic interaction [80].

#### 3.4.4. Ex Vivo 

Ex vivo mucoadhesion strength was determined by using texture profile analyzer. Mucin tablet was adhered to the lower and upper probe of the texture analyzer using double sided adhesive tape. Polymer tablet was hydrated in simulated gastric fluid (SGF) having pH 1.2. Further, the hydrated polymer tablet was placed over the lower mucin tablet (attached with lower probe) and the upper probe was moved downwards to bring mucin tablet in contact with hydrated polymer tablet kept over the lower mucin tablet. The downward force was applied to provide intimate contact between the polymer tablet and the mucin tablet. After 1 min, the upper probe was move upward at constant speed (0.5 mm/s), the detachment force of the mucin tablet from the polymer tablets was determined by the force vs. time plot. On the other hand, the mucoadhesion time was measured by placing the tablet on the mucosal layer and tied to the side of the paddle of dissolution apparatus. The paddle was then rotated at 50 rpm containing phosphate buffer pH 6.8 and temperature 37 °C. The time when the tablet was separated from the intestinal mucosal layer was recorded as mucoadhesion time [26,28]. Thirawong et al. analyzed mucoadhesive properties of various pectins on gastrointestinal mucosa using texture analyzer and studied various parameters and test conditions such as contact time, test speed of probe withdrawal, pre-hydration time of pectin disc, contact force, test medium, GI tissue, work of adhesion (W_ad_) and maximum detachment force (F_max_). He concluded that the degree of hydration of pectin disc may affect the mucoadhesion properties of pectin and the mucoadhesive strength of pectin was increased with an increase in contact force and contact time but not by the withdrawal speed of probe. He also revealed that F_max_ and W_ad_ showed much higher values in pH 4.8 incomparison to pH 1.2 [81]. 

## 4. Mechanical Properties

Mechanical properties are used to denote stress–strain relationship of the polymeric system. Some examples of such mechanical properties include tensile strength of the material, fatigue limit of the material, modulus of elasticity of that material, hardness limit of the material, elongation value of that material and many more [82]. Some basic mechanical properties of polymer or any material are mentioned below:

### 4.1. Percent Elongation

It is defined as the percentage measure representing the change in length of the polymeric substance or of any material. It is total amount of pressure required by the material for its breakage. It is also a measure of ductility of the polymer [83,84].

### 4.2. Young’s Modulus

Young’s Modulus is defined as the ratio of stress and strain of the material. Stiffness of the material is a measure of elastic modulus [85,86].

Young’s Modulus(E) = Tensile Stress(σ)/Tensile Strain(ε)

### 4.3. Toughness

The total area under a stress–strain curve is mentioned as toughness of a material. Mathematically, it can be explained as:

Toughness = ∫σdε

The total energy required to break the material is the representative of toughness of material. Stress–strain curve represents the behavior of various materials or polymeric materials when being applied under different stress–strain conditions. High modulus of elasticity possesses brittle and hard polymeric materials. Ductile polymeric materials/ substances also have high Young’s Modulus value. The toughness of the polymeric materials is inversely proportional to temperature. With an increase in the temperature, the toughness of the material reduces however reduction in the temperature make the material more rigid, compact and harder resulting in increase in toughness. Moreover, it may also affect the mechanical properties of the polymers as one study revealed that chemical structure and crosslinking density of methacrylate shape memory polymer networks effects the thermo-mechanical properties and toughness of the polymer. The T_g_ (glass transition temperature) of (meth)acrylate networks increases by adding α-methyl groups and moving bulky side groups close to the backbone of the polymer [87,88].

### 4.4. Viscoelasticity

Let us consider the constant stress applied to any polymeric material. In case of elastic deformation, the material comes back to its initial conditions. When the strain is applied on the material some polymeric changes occur in the material. However, when we remove the stress from the material, the material regains its original condition making the transformation reversible [89,90]. Mathematically it can be explained as:

σ = Eε

where, E is the representative of elastic modulus, σ is the representative of applied stress, ε is the representative of strain developed.

On the other hand, when the strain on the polymeric material keeps on increasing with time then the recovery process is delayed. However, if the strain continues, then the material does not return to initial state making the changes irreversible [91,92]. Mathematically it can be explained as:

σ = γdε/dt

where, γ is the representative of viscosity, dε/dt is the representative of strain rate.

**Figure 4:**
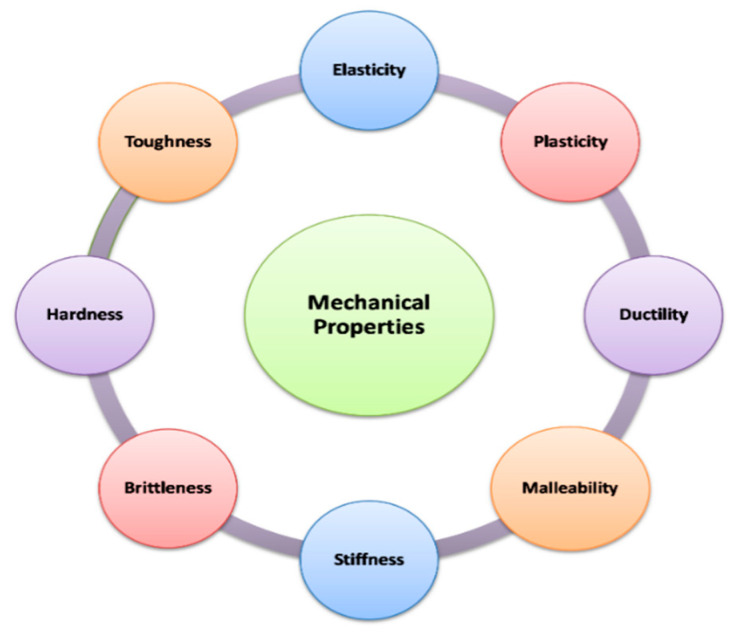
Indicators of mechanical properties of biopolymers.

Generally, a combined behavior of polymer (plastic and elastic deformation) depends upon the strain rate and temperature (Figure 4). At low strain rate and high temperature, the viscous behavior of polymer is observed and an elastic behavior is observed when there is high strain rate and low temperature. The combined behavior (viscosity and elasticity) is observed at transitional temperature and strain rate values. This behavior is termed as viscoelasticity, and the polymer is termed as viscoelastic in nature [93,94].

Thiolation of poly vinyl alcohol (PVA) by esterification with 3-mercaptopropinic acid was performed and the thiolated PVA (TPVA) and wheat gluten (WG) blends exhibited larger strain at break and flexure strength with higher modulus as compared to pristine PVA/WG blends. Mechanistically, -SH (thiol) functionalities in TPVA support multiple disulfide-sulfhydryl exchange reaction which alters the distribution of inter and intra molecular disulfide linkages responsible for enhancement of mechanical properties [95]. Similar enhancement in mechanical properties was reported in wheat gluten modified with thiolated poly (ethylene oxide) [96].

Thiolation of chitosan and polycarbophil using thioglycolic acid and L-cysteine was carried out, respectively. These thiolated polymers were used for developing vaginal gels for treating human papilloma virus infections increase in thiol conjugation resulted in the enhancement of cohesiveness, elasticity and mucoadhesion of the gel formulation. Polycarbophil and its thiol conjugate prolonged the release of drug for more than 72 h. As compared to gel formulation of chitosan and thiolated chitosan which released drug upto 9–12 h [97]. On the other hand, Cevher and co-workers in 2008, prepared conjugates of polyacrylic acid and cysteine for formulation of vaginal gels of clomiphene citrate. Carbopol 934P, Carbopol 971P and carbopol 974P were used in the study. Gel formulation of Carbopol 934P and its conjugates exhibited appropriate compressibility and hardness for its application to vaginal mucosa and showed good cohesion and spreadibility for preventing disintegration of gel in the vaginal mucosa [98].

Methacrylate thiol-ene as dental restorative materials were prepared. The incorporation of thiol-ene mixtures into dimethacrylate resins reduced frexural strength (by 6–20%) and shrinkage stress (by 5–33%). The combination of reduced shrinkage stress and good flexural properties were advantageous in dental restorative materials application [99].

Self-healing dynamic hydrogels of mixture of Au-thiolate and disulfide bonds were prepared. The mechanical properties of Au based hydrogels could be tuned by varying the concentration of 4 arm thiol terminated polyethylene glycol and the concentration of thiolated Au incorporated in the hydrogel network. Such materials could be used as visco-supplementation materials and also as scaffolds for drug/cell delivery [100].

Podgorski et al. executed conventional crosslinking reactions by synthesizing tetra (2-mercaptoethyl)silane. The ester free thiol-ene materials revealed enhanced mechanical properties such as elasticity modulus, elasticity and toughness. These ester free thiol-ene materials are ideal candidates for various medical applications such as lithography, coating and dental resin applications [101]. Rakas et al. synthesized norbornene and norbornene siloxane predecessor using thiol (multifunctional) as crosslinking agent. The crosslinker was used to produce non-acrylte (UV curable adhesive system). The added filler extensively increased the mechanical parameters (tensile stress and strain properties) of the material. In addition, the type and concentration of filler also influences thiolated polymer thermal properties. As the filler concentration increased concentration, melting temperature also increases and films were produced with widened cold crystallization range [102].

## 5. Mucoadhesive Properties and Dosage Forms

Mucoadhesive properties play a significant role in the development of novel mucoadhesive (bioadhesive) delivery systems and in screening of materials and their mechanisms. There are several methods which have been developed to analyze mucoadhesive properties i.e., mucoadhesion. As no standard apparatus or instrument has been designed for the evaluation of bioadhesive strength, some researchers have employed texture analyzer and modified analytical balance method for determination of bioadhesive strength. Furthermore, there are three testing modes which are identified such as peel strength, shear strength and tensile analysis for the measurement of mucoadhesive properties [103]. Grobovac et al. discussed mucoadhesive properties of thiomers and compared with other established polymers. The mucoadhesive properties (tensile study, shear strength, peel strength) of tested polymers were improved irrespectively both in case of anionic thiomers (2-fold and 20-fold) and cationic thiomers (100-fold and 140-fold). Moreover, the chemical parameter i.e., polymeric molecular weight, also showed a great impact on mucoadhesive properties of thiomers, resulted that molecular mass was indirectly proportional to mucoadhesive properties (lesser the molecular mass, the more mucoadhesive properties) [104].

### 5.1. Tablets

**Figure 5:**
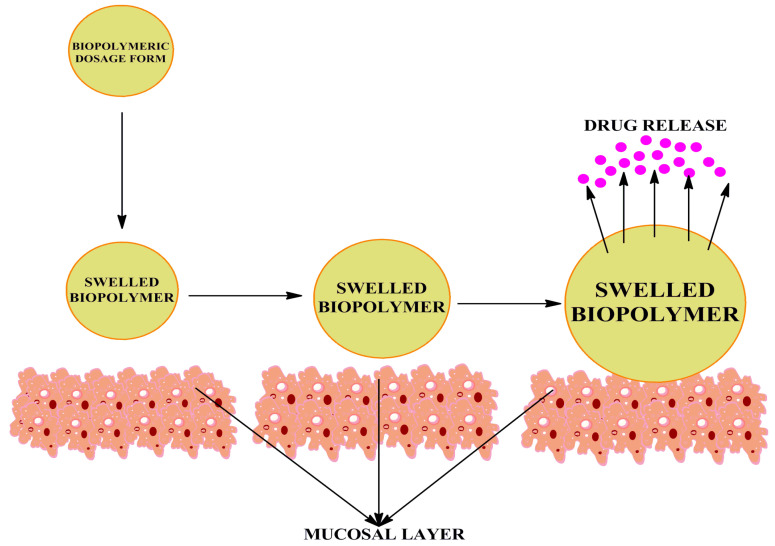
Illustrates swelling behavior of biopolymeric dosage form (tablet) when it enters the GI tract.

Biopolymeric dosage form (tablet) is an oral solid dosage form which can be easily formulated by adding number of excipients (biopolymers) with one or more active drug. Tablets may differ in physical properties (shape, size, hardness, thickness and weight), dissolution and disintegration characteristics and in other aspects such as the manufacturing method and intend use. The most extensively accepted general mechanism of action for tablet disintegration is swelling (Figure 5) [105,106]. Millotti et al. synthesized chitosan-6 mercaptonicotinic acid for developing sustained release tablets for oral delivery of insulin. Thiolated polymer was proven to be a promising material for the systemic delivery of insulin and other peptide drugs [107]. Baloglu et al. prepared mucoadhesive tablets using poly acrylic acid and cysteine conjugates for the vaginal delivery of econazole and miconazole nitrate. Significant mucoadhesive property with sustained drug release was reported with the use of thiolated polymer [108]. Madgulkar et al. prepared vaginal mucoadhesive tablets of clotrinazole using thiolated xyloglucan. Improved antifungal activity of the drug along with sustained release was reported in the work [109]. Naveen et al. performed thiolation of okra gum for developing mucoadhesive tablets of repaglinide. Enhanced swelling, mucoadhesive strength and sustained drug release was reported in the study [110].

### 5.2. Films

Polymeric films are mainly used to coat tablets dosage forms but somehow they also act as dosage form such as transdermal films, buccal films, ophthalmic films, nasal films and so on. Films rapidly swell and disintegrate into body fluids and the incorporated drug is released immediately [111]. Jalil et al. performed conjugation of gellan gum with 2(2aminoethyl disulfanyl)nicotinic acid and prepared mucoadhesive vaginal films for the delivery of metronidazol S-protected gellan gum exhibiting 1.84- to 4.3-fold increase in dynamic viscosity in porcine mucus and 3-fold increase in mucoadhesive property [112]. Zaman et al. evaluated thiolated arabinoxylan sustained release mucoadhesive polymer. Thiolated polymer confirmed 6.01 ± 1.03 m moles of thiol groups per gram of the polymer. A Mucoadhesive buccal film for the delivery of tizamidine was developed [113]. Naz et al. performed thiolation of sodium carboxymethyl cellulose and chitosan with thioglycolic acid and cysteine. Buccal films for the delivery of fluconazole were developed. Thiolated films exhibited 5.8-fold higher mucoadhesive properties compared to unmodified films [114]. Hanif and Zaman developed oral mucoadhesive films of tizanidine using thiolated arabinoxylan. Films were reported to possess significant mechanical strength and mucoadhesive property. [115]. Ahmad et al. fabricated microneedle patches of tacrolimus employing thiolated chitosan. The transdermal patch demonstrated good mechanical properties with improved bioavailability and sustained drug release behavior [116].

### 5.3. Fibers

Biopolymeric fibers have been extensively used in drug delivery systems. Various thiolated polymeric fibers can be employed as a controlled mucoadhesive drug delivery system [117,118]. Nanofiber mats of polycaprolactone were prepared using 1-propanethiol as monomer by electrospining method under low pressure plasma polymerization technique. Thiol rich polycaprolactone nanofibers were proven to be a potential candidate for tissue engineering applications [119]. Dong et al. performed rheology and electrospinning of a series of wheat gluten mixtures with poly(vinyl alcohol), dithiothreitol and thiolated poly(vinyl alcohol) in water/1-propanol [120]. Shanmuganathan et al. reported preparation and characterization of stretchable thermoset fibers using thiol-ene polymerization technique. Fibers were derived by in situ photopolymerization of tetrafunctional thiol and trifunctional vinyl ether monomer. Nonwoven mats exhibited much higher elongation and stretchable at a break of 85%. These stretchable fibers show potential applications as hot chemical filtration, composite material, textile and biomedical [121]. Polatet al. developed besifloxacin loaded nanofibrous ocular inserts of poly(caprolactone) and polyethylene glycol followed by coating with thiolated sodium alginate. Coating with thiolated polymer resulted in reduced frequency of application leading to increased patient compliance [122].

### 5.4. Nanoparticles

Nanoparticles are a wide class of materials which include particulate substances that have one dimension less than 10 nm. Biopolymeric nanoparticles are the existing carriers for the targeted and controlled drug delivery systems for various natural and synthetic drugs. These nanoparticles are compatible with the biological environment and improve the mucoadhesion with mucous membrane and cellular penetration. Menzel et al. prepared mucoadhesive nanoparticles after pre activation with 2-mercaptonicotinic acid by ionic gelation method with polyethylenimine. Thiolated particles showed an increase in viscosity as compared to unmodified one. Furthermore, preactivated polymers showed better improvement in mucoadhesive properties [123]. Esquivel et al. derivatized low molecular weight thiolated chitosan by a coupling reaction with 3-mercaptopropionic acid. Further, thiolated chitosan nanoparticles were formulated using high concentration of sodium tripolyphosphate. The optimized semispherical nanoparticles of thiolated chitosan were synthesized with the parameter of pH 4.7 and molar ratio 1:106. The results were promising for possible application of nanoparticles as nanocarrier and delivery systems [124]. Saremi et al. prepared core shell nanoparticles of thiolated chitosan coated on polymethyl methacrylate as a carrier using docetaxal as therapeutic agent. Nanoparticles prepared with thiolated chitosan exhibited more cytotoxic effect in cancer cell as compared to free drug after 72 h [125]. Sudhakar et al. studied biodistribution and pharmacokinetic profile of insulin from thiolated chitosan nanoparticles. Significant prolonged release and improved bioavailability of insulin was reported due to enhanced mucoadhesive interactions of the thiolated chitosan with the mucosal surfaces [126].

### 5.5. Gels/Hydrogels

Hydrogels are three dimensional hydrophilic polymer networks and are capable to swell/spread in water and biological fluids (saliva). There are various types of hydrophilic groups (–OH, –COOH, –CONH_2_, –CONH, –SO_3_H, etc.) present in hydrogels through which hydrogels are able to absorb water. The controlled and release mechanism of the drug from the hydrogels can be modified by adjusting the factors like water content, polymer composition, crystallinity and crosslinking density. Gajendiran et al. developed thiolated polymeric hydrogels for tissue engineering applications. Thiolated carbohydrate based biopolymers, thiolated protein based biopolymers and thiolated synthetic polymers were used to fabricate hydrogel matrices. Some thiolated carbohydrate polymers (heparin, pectin, collagen, gelatine, polygalacturonic acid) were synthesized and hydrogels were prepared via cross-linking reaction. Prepared hydrogels showed better mucoadhesive properties with respect to that of thiol free polymers [127]. Asim et al. synthesized S-protected thiolated hyaluronic acid and reported it to be stable towards oxidation and forms highly cohesive gel after contact with endogenous thiols, demonstrating its potential for 3D cell culture scaffolds [128].

Self-healing thermo-responsive hydrogel injectables were prepared in 2017 by Yu and his team. Dynamic hydrogel by thiol/disulfide exchange reaction was formed by simple mixing of thiol functionalized F127 and dithiolene modified PEG. Prepared hydrogels self-heal not only under alkaline conditions but also at neutral or even mildly acidic conditions due to increase in the reactivity of disulfide of cyclic dithiolene [28,129,130].

## 6. Conclusions

Biopolymers are found abundantly in nature. These are cheaper, biocompatible, nontoxic due to which they are extensively used in various capacities in pharmaceutical product development. Ease of modification is another advantageous element stimulating the researchers to develop different modified versions of biopolymers with customized physicochemical properties. Incorporation of thiol group into the molecular structure of the biopolymers leads to enhanced mucoadhesive and mechanical properties. Different agents employed for the thiolation of biopolymers like Traut’s reagent, dithiothreitol reduction, dithiolaromatic, thiol polyethylene glycolamine and thioglycolic acid are reported in this paper. The thiolated biopolymers could be characterized for degree of thiolation, swelling behavior, mucoadhesion and mechanical properties. Various instrumental techniques like FTIR, DSC, NMR, XRM and SEM could be employed for characterization and elucidation of thiolated biopolymers. Different drug delivery systems viz. tablets, films, fibers, nanoparticles, microparticles, etc. could be developed employing thiolated polymers for drug delivery to different region of the body. For large scale development, reproducibility, quality and properties of thiolated polymers are important points of concern. Toxicity, residual content, impurities and regulation issues are some other critical issues that should be handled prior to commercial exploitation of thiolated biopolymers.

## Figures and Tables

**Table 1:** Origin, chemical structure and properties of polymers.

Polymer	Source	Solubility	Charge	Structure
Gelatin	Animal collagen of bones, tendons and skin	Soluble in water or some alcohol	Positive or negative (the isoelectric point depends on its extraction procedure from collagen)	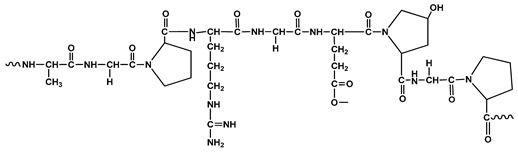
Hyaluronic acid	Connective, epithelial and neural tissues	Soluble in water, Slightly soluble in organic solvent	Negative charge	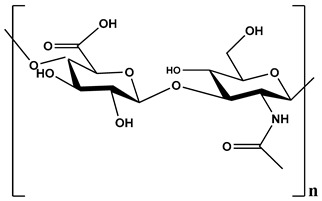
Chitin	Exoskeletons of arthropods, shells of crustaceans and cell walls of yeast and fungi	Dilute acidic medium	Positive charge	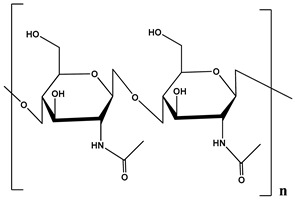
Alginate	Cell wall of brown seaweed	Water Soluble	Negative charge	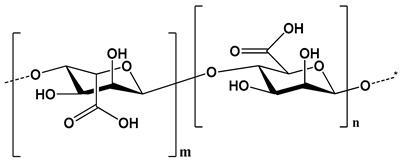
Pectin	Inner rind of citrus peel (Cirtusaurantium)	Soluble in hot water	Negative charge	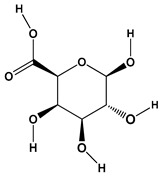
Carrageenan	Red seaweed (also called Irish moss)	Iota and kappa sodium salts are soluble in water at 20 °C	Negative charge	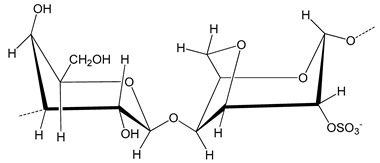
Karaya gum	Stems and branches of strains of: Sterculia urens (Roxburgh) and other species of Sterculia	Soluble in alkali solvents	Positive or negative (ionic charge)	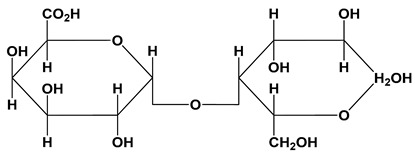
Xanthan gum	Derived by fermentation of Gram-negative bacteria Xanthomonas campestris	Soluble in both cold and hot water	Negative charge	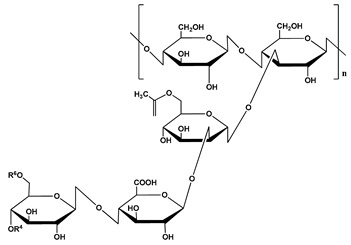
Psyllium mucilage	Seed coat of Planta goovata	Soluble in water	Negative charge	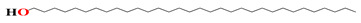
Gellan gum	Produced by the bacterium *Sphingomonas elodea*	Soluble in water	Negative charge	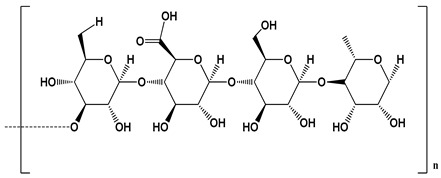
Moringa gum	Oils are made from the seeds, while powders can be made from the leaves and roots	Soluble in water	Negative charge	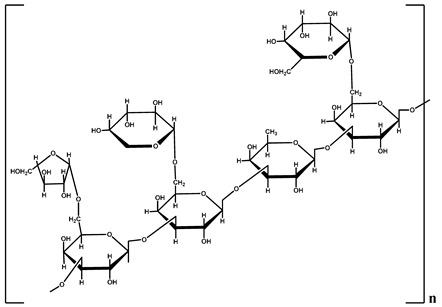

**Table 2:** Reported literature on thiolation of biopolymers for developing mucoadhesive formulations.

Sr. No.	Biopolymer	Thiolation Moiety	Thiolation Formulation	Remarks	Reference
1.	Gelatin	Traut’sreagent (2-Iminothiolane)	Hydrogel	✓ Hydrogels are flexible, transparent, strong and crosslinking is reversible	[13]
2.	Gelatin	l-cysteine	Hydrogel	✓ Hydrogel showed lower cumulative gelatin dissolution, higher water content, maximum weight and swelling ratio ✓ Fibroblasts encapsulated within the hydrogel (crosslinked) showed formation of cellular network	[14]
3.	Hyaluronic acid	Dithiobis (propanoic dihydozide) and Dithiobis(butyric dihydrozide)	Hydrogels	✓ The disulfide cross-linking was reversible ✓ In-situ encapsulation of murine fibroblasts during gelation of HA-DTPH ✓ pka = 8.87 and 9.01 for (HA-DTPH and HA-DTBH)	[15]
4.	Hyaluronic acid	l-cysteine	Tablets	✓ The mucoadhesive properties increased by 6.5-fold ✓ Formation of inter and intra molecular disulfide bonds ✓ Non-Fickian kinetic and showed sustained release for <12 h	[16]
5.	Chitosan	3-mercaptopropionic acid	Nanoparticles	✓ Prepared nanoparticles are stable, semispherical ✓ Not aggregated at high concentration of thiamine pyrophosphate	[17]
6.	Chitosan	Traut’s reagent (2-Iminothiolane)	Microparticles	✓ Prepared microspheres are more stable in aqueous media. ✓ Able to control the drug release for about 1 h. ✓ Showed increase in 2.5-fold in concentration of chitosan-TBA conjugate microparticles	[18]
7.	Alginate	Cysteine hydrochloride monohydrate	Tablets	✓ 2-fold increase in detachment force of the tablet ✓ Less toxic to L-929 mouse fibroblast cell line	[19]
8.	Alginate	Cysteine methyl ester hydrochloride	Hydrogel	✓ Excellent biocompatibility and appropriate mechanical strength ✓ Time to hemostasis of rat reduced from 8.26 min to 3.24 min	[20]
9.	Pectin	Thioglycolic acid	Beads	✓ Improved the mucoadhesive potential of pectin without affecting the release profile	[21]
10.	Pectin	4-Aminothiophenol	Hydrogels	✓ Showed coupling of 4-aminothiophenol with pectin by 557.3 ± 49.0 and 158.8 ± 23.1 ✓ Rheological study showed 500-fold increase in viscosity	[22]
11.	Carrageenan	Thiourea	Tablets	✓ Increase in cell treated with thiolated κ and ι- carrageenan (1.38- and 1.35-fold) ✓ Increase in dynamic viscosity of mucus with modified κ and ι- carrageenan (3.9- and 2.0-fold) ✓ Residence time of modified κ and ι- carrageenan on porcine intestinal mucus was 6.4- and 1.8-fold prolonged indicating improved mucoadhesion	[23]
12.	Karaya Gum	Thioglycolic acid	Tablets	✓ Formation of disulfide bonds showed enhanced mucoadhesion, swelling of tablets and sustained release ✓ Showed increase in pH from 1.2 to 6.8	[24]
13.	Xanthan Gun	Thioglycolic acid and mercaptopropionic acid	Buccal Pellets	✓ Modified Xanthan gum improves its mucoadhesive properties	[25]
14.	Psyllium Mucilage	Thioglycolic acid	Gel	✓ Showed an increase in the mucoadhesive strength and mucoadhesion retention time by 3-fold and 1.5-fold	[26]
15.	Gellan Gum	Thioglycolic acid	Gel	✓ Showed greater adhesive strength on goat intestinal mucosa (3.75-fold)	[27]
16.	Moringa Gum	Thioglycolic acid	Tablets	✓ Having 0.956 ± 0.024 mM of thiol groups ✓ Higher ex vivo bioadhesion time (1.5-fold) ✓ Sustained release of metronidazole over 24 h	[28]

**Table 3:** Patents filed by researches on thiolated biopolymers.

Patent Number	Title	Inventor	Original Assignee	Reference
US8124757B2	Thiol-modified macromolecule derivatives and cross-linked materials thereof	Chan Song	Bioregen Biomedical (Chang zhou) Co., Ltd.	[40]
US9895394B2	Induction of chronic elevation of intraocular pressure with vinysulfonated hyaluronic acid (HA-VS) and thiolated hyaluronic acid (HA-SH)hydrogel	Kai-shun Christopher Leung, Ying Chau, Yu Yu	Kai-shun Christopher Leung, Ying Chau, Yu Yu	[41]
US10137199B2	Thiolated hyaluronan-based hydrogels cross-linked using oxidized glutathione	Thomas Zarembinski, Isaac Erickson, Nathaniel Doty	BioTime Inc.	[42]
US9198997B2	Rehydratable thiolated polysaccharide particles and sponge	Matthew Franco Myntti, Dana A. Oliver, Brian Vaccaro	Medtronic Inc.	[43]
US3914214A	Thiolation of polysaccharides	Donald Trimnell, Baruch S Shasha, William M Doane	US Department of Agriculture	[44]
US3007918A	Thiolation of carbohydrates	Benesch Reinhold, Ruth E Benesch	Research Corp.	[45]
US20060135585A1	Compounds and methods for thiol-containing compound efflux and cancer treatment	Brian J. Day, Remy Kachadourian	National Jewish Health Co.	[46]
US3574820A	Medicinal dosage forms of unpolymerized thiolated gelatin with a cross-linking accelerating agent providing slowly released medication from a swollen matrix	Richard H Johnson, Englebert L Rowe	Upjohn Co.	[47]
US20120283467A1	Method for preparing polyols by means of thiolation and products such as those obtained	Henri Cramail, Aurelie Boyer, Eric Cloutet, Carine Alfos	Centre National de la Recherche Scientifique CNRS	[48]
CN105688284B	In-situ hydrogel capable of imitating extracellular matrix injection and preparation method and application thereof	Meng, T.; Chao, Y.	-	[49]
US6884788B2	Thiol-modified hyaluronan	Paul C. A. Bulpitt, Charles H. Sherwood, Khalid K. Sadozai	Anika Therapeutics Inc.	[50]
WO2008008857A3	Thiolated macromolecules and methods of making and using thereof	Glenn D. Prestwich, Monica Serban	Glenn D. Prestwich Monica Serban Univ Utah Res Found	[51]
US4699950A	Block copolymer based on polymer having thiol end group and linked by divalent sulfur	Toshiaki Sato, Junnosuke Yamauchi, TakujiOkaya	Kuraray Co., Ltd.	[52]
US10189952B2	Degradable thiol-ene polymers	Christopher Bowman, Kristi Anseth, Bilge Hacioglu, Charlie Nuttelman	University of Colorado Boulder	[53]
US9492381B1	Method of administering hyaluronan formulation for preventing and ameliorating osteoarthritis	James D. Smith	Bi Investment LLC	[54]
US7465766B2	Hydroxyphenyl cross-linked macromolecular network and applications thereof	Anthony Calabro, Lee Akst, Daniel Alam, James Chan, Aniq B. Darr, Kiyotaka Fukamachi, Richard A. Gross, David Haynes, Keiji Kamohara, Daniel P. Knott, Hilel Lewis, Alex Melamud, Anthony Miniaci, Marshall Strome	Cleveland Clinic Foundation	[55]
US8980295B2	Multifunctional in situ polymerized network via thiol-ene and thiol-maleimide chemistry	Weiyuan J. KAO, Yao Fu	Wisconsin Alumni Research Foundation	[56]
AU2018201206	Locally released growth factors to mediate motor recovery after stroke	Stanley T. Carmichael, Andrew N. Clarkson, Michael D. West	University of California Biotime Inc.	[57]
